# Neutrophil-to-lymphocyte ratio predicts long-term survival in early triple negative breast cancer treated with neoadjuvant chemotherapy

**DOI:** 10.3332/ecancer.2025.2034

**Published:** 2025-11-14

**Authors:** Gabriel Berlingieri Polho, Leticia Kimie Murazawa, Vinicius Vitor Oliveira, Victor Rocha Pinheiro, Diana del Cisne Pineda Labanda, Yumi Ricucci Shinkado, Romualdo Barroso-Sousa, Luciana Rodrigues Carvalho Barros, Laura Testa, Renata Colombo Bonadio

**Affiliations:** 1 Instituto do Câncer do Estado de São Paulo, Av Dr Arnaldo, 251 - Cerqueira Cesar, São Paulo, SP 01246-000, Brazil; 2 Dasa Oncology, Brasilia Hospital, SHIS QI 15 - Lago Sul, Brasília 71635-240, Brazil; 3 Instituto D’Or de Pesquisa e Ensino (IDOR), Av Brigadeiro Luís Antônio, 5001 - Jardim Paulista, São Paulo, SP 22281-100, Brazil; a https://orcid.org/0000-0001-5818-922X

**Keywords:** neutrophil-to-lymphocyte ratio, triple-negative, breast cancer, neoadjuvant

## Abstract

**Purpose:**

Biomarkers for tailoring treatment in neoadjuvant triple-negative breast cancer (TNBC) are needed. We hypothesize that neutrophil-to-lymphocyte ratio (NLR) before neoadjuvant chemotherapy (NACT) can predict long-term outcomes in this population.

**Methods:**

We reviewed our institutional database to identify patients with clinical stages II–III TNBC who underwent NACT from 2012 to 2024 and retrospectively collected data from medical records. We calculated event-free survival (EFS) from the date of NACT initiation until death, disease recurrence or disease progression that precluded surgery; we calculated overall survival (OS) from the date of NACT initiation until death. Survival estimates were analysed using Kaplan–Meier method and compared with log rank test. The Cox regression model was used to calculate hazard ratios.

**Results:**

A total of 692 patients were included in the analysis. Of these, 63.3% had stage III disease, 60.8% had grade 3 tumours and 77.2% had a Ki-67 >50%. The most common NACT regimen used was anthracycline and taxane-based (96.8%). The overall pathological complete response (pCR) rate was 27.7%. After median follow-up of 59.6 months, NLR >2 was associated with poorer EFS (HR 1.71, 95% CI 1.33–2.18, p < 0.001) and OS (HR 1.76, 95% CI 1.34–2.31, p < 0.001). The results maintained statistical significance after adjusting for age, ki67, clinical stage and pCR status (p = 0.002).

**Conclusion:**

NLR predicts long-term survival after NACT in TNBC and, as a readily and inexpensive information, should be further studied in current approaches of chemoimmunotherapy.

## Introduction

Triple-negative breast cancer (TNBC) has a higher risk of recurrence and poorer survival outcomes compared to other breast cancer subtypes, due to its aggressive nature and the lack of targeted therapies until recent years. Traditional treatment options for TNBC have primarily relied on chemotherapy, but advances in immunotherapy and antibody drug-conjugates are improving some patients' outcomes [1, 2].

The incorporation of immune checkpoint inhibitors as part of neoadjuvant therapy has sparked significant interest in identifying reliable biomarkers that can better stratify patients. Furthermore, selecting adequately the patients who are most likely to benefit from immunotherapy could be specifically useful in resource-limited scenarios.

One potential biomarker is the neutrophil-to-lymphocyte ratio (NLR), an easily measurable parameter derived from routine blood tests. NLR is considered an indicator of systemic inflammation, and elevated levels have been associated with worse prognosis in several malignancies, including breast cancer [3, 4].

In TNBC, some studies suggest that higher NLR is associated with worse survival outcome [4, 5] and lower response rates to neoadjuvant chemotherapy (NACT) [6]. The role of NLR as a potential prognostic and predictive biomarker in this context warrants further investigation, particularly in relation to its ability to stratify patients according to their likelihood of achieving favourable outcomes after chemotherapy.

## Methods

We retrospectively reviewed our institutional database to identify female patients with early-stage TNBC who were treated with NACT between 2012 and 2024. Inclusion criteria were: (1) female patients with histologically confirmed TNBC; (2) initiation of NACT; and (3) clinical stages II or III disease. Exclusion criteria were: (1) metastatic disease at diagnosis; (2)] immunohistochemistry expression of estrogen receptor (ER) or progesterone receptor >1%; (3) phyllodes tumour; (4) synchronous bilateral breast cancer; (5) second primary tumour (except localised non-melanoma skin cancer diagnosed within the past 5 years); and (6) patients with incomplete medical records or unavailable data in the electronic medical charts. Approval from the local ethics committee was obtained prior to data collection (Access Number: 76319823.3.0000.0068).

### Outcomes and statistical considerations

NLR was calculated using absolute neutrophil and lymphocyte counts in the initial blood count before NACT initiation. The cutoff point used in the study was NLR of two based on previous literature, distribution of NLR in our cohort and a pilot study previously presented from our group [7].

Event-free survival (EFS) was defined as the time from NACT initiation until death, disease recurrence or disease progression that precluded surgery. Overall survival (OS) was defined as the time from NACT initiation until death. Patients without the events were censored at the time of last follow-up.

Pathological complete response (pCR) was defined as ypT0/ypTis and ypN0 in surgical specimens. If the patient had disease progression during NACT or toxicities that precluded surgery, the patient was considered as non-pCR. Four patients were included in a breast-conserving protocol and allocated to no surgery after complete image response and were excluded in the analysis related to pCR.

Logistic regression was used to assess the correlation between pCR and other independent variables, in univariate and multivariable models.

Survival estimates were analysed using Kaplan–Meier method and compared with log rank test. The Cox regression model was used to calculate Hazard Ratios in univariate and multivariable analysis. Chi-squared test was used to compare categorical variables and Mann–Whitney test to compare numerical variables. Comparison of numerical variables between more than two groups were made with Kruskal–Wallis test.

## Results

### Patients characteristics

**Figure 1.**
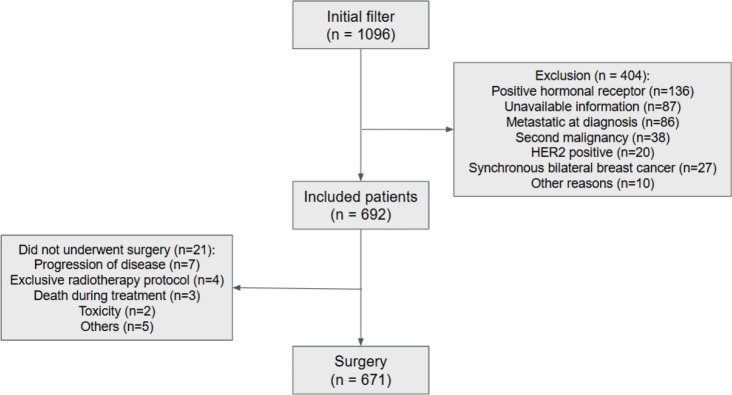
CONSORT.

Among the 692 patients included (Figure 1), 63.3% had stage III disease, 60.8% grade 3 disease and 77.2% ki67 ≥50%. Median age was 48.5 years and the most common NACT regimen used was anthracycline and taxane-based (96.8%). Patients with high NLR were younger, had an increased proportion of stage III disease and larger tumours, as shown in the distribution of Table 1. Median NLR was not different among races (*p* = 0.16).

### Outcomes

The overall pCR rate was 27.7% and pts with NLR >2 had an decreased probability of achieving pCR (22.9% versus 32.9%, *p* < 0.01).

The univariate logistic regression showed association of NLR, ki67 and clinical stage with pCR, which remained significant in the multivariable model including these variables (Table 2).

After a median follow-up of 59.6 months, NLR >2 was associated with poorer 5-year EFS in the overall population (51% versus 66% HR 1.71, 95% CI 1.33–2.18, *p* < 0.001).

**Figure 2.**
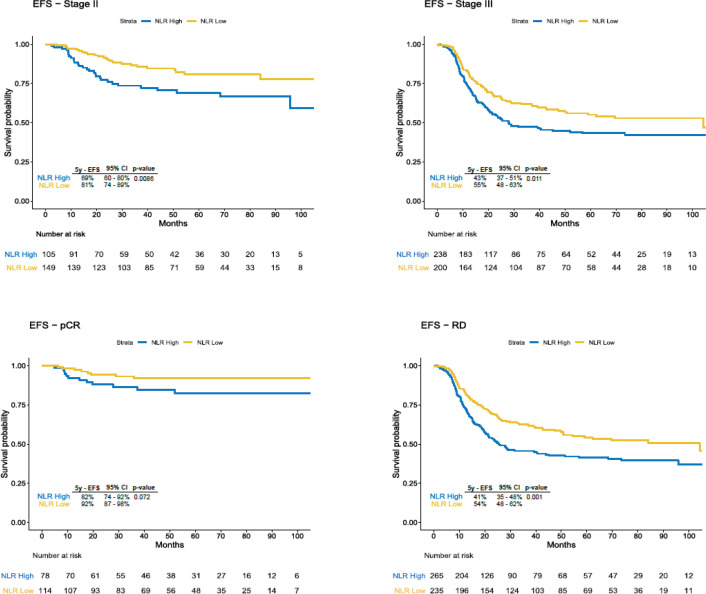
EFS according to subgroups. EFS: Event-Free Survival; 95% CI: 95% Confidence Interval; pCR: pathological complete response; RD: Residual Disease.

**Figure 3.**
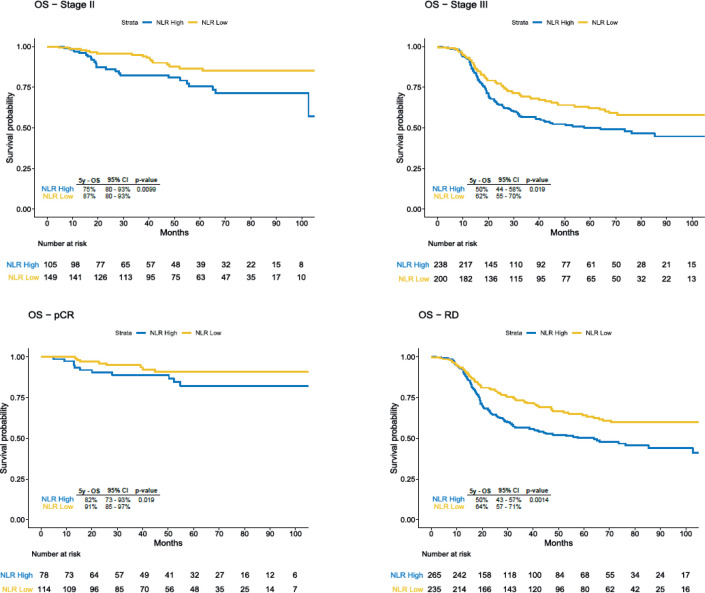
OS according to subgroups. OS: Overall Survival; 95% CI: 95% Confidence Interval; pCR: pathological complete response; RD: Residual Disease.

Due to the disproportion in stage III disease and pCR between categories of NLR, we stratified the analysis according to the clinical stage and pCR status. We observed significant difference in EFS in the subgroup of patients with stage II disease (69% versus 81%, HR 2.03, 95% CI 1.18–3.47, *p* = 0.008), stage III (43% versus 55%, HR 1.43, 95% CI 1.08–1.89, *p* = 0.01), residual disease (41% versus 54%, HR 1.54, 95% CI 1.19–1, *p* = 0.001) and a trend for worse survival rates for patients with pCR and high NLR (82% versus 92%, HR 2.23, 95% CI 0.91–5.54, *p* = 0.07) (Figure 2). 5-year OS was also inferior in the overall population with NLR >2 (58% versus 73%, HR 1.76, 95% CI 1.34–2.31, *p* < 0.001), in stage II disease (75% versus 87%, HR 2.26, 95% CI 1.20–4.29, *p* = 0.009), in stage III disease (50% versus 62%, HR 1.44, 95% CI 1.06–1.95 *p* = 0.02) and in those with residual disease (50% versus 64%, HR 1.59, 95% CI 1.19–2.13, *p* = 0.001), but in patients who achieved pCR the difference was not statistically significant (82% versus 91%, HR 2.00, 95% CI 0.80–4.98, *p* = 0.1) (Figure 3).

For multivariable analysis, we included age, pCR status, clinical stage, ki67 and NLR. These variables were chosen because of imbalanced datasets between high and low NLR subgroups and potential interactions between them. In multivariate analysis, including pCR status, clinical stage and age (≥40 versus < 40), NLR >2 remained associated with worse OS (*p* = 0.002) and EFS (*p* = 0.002) (Table 3).

When exploring different cut-off points, using values reported elsewhere [4, 7], similar results were found (Supplementary Tables 1 and 2). Interestingly, for those who achieved pCR and had an initial NLR ≤1.43, 5 year EFS was 98%.

## Discussion

The incorporation of immunotherapy in the treatment landscape of early TNBC has improved survival outcomes at the expense of increased physical and financial toxicities [2]. Therefore, research for potential predictive and prognostic biomarkers has become important to appropriately select patients to escalate or de-escalate treatment.

NLR is a simple marker that has been previously implicated as a potential prognostic and predictive biomarker after NACT for early breast cancer. One meta-analysis, including more than 8,000 patients with different subtypes of breast cancer and clinical stage, found that NLR is associated with worse OS and disease-free survival, and it was more pronounced in ER negative and/or HER2 negative [4]. Another meta-analysis also showed that low NLR is associated with higher pCR rates, but no specific analysis was conducted in patients with TNBC [8]. Our study is in line with previous literature: our data suggests that higher NLR before NACT is associated with more advanced disease and is an independent marker of poorer prognosis in the overall cohort, as well as within subgroups of stage II disease, stage III disease and residual disease. There was also a non-statistically significant difference of prognosis for those who achieved pCR.

To explore the influence of NLR on survival and considering the imbalance between NLR high versus low groups and possible interactions between variables, we performed multivariable regression models. NLR, clinical stage and pCR status were significantly associated with survival in the multivariable model. Interestingly, ki67 was only associated with survival when included in the multivariable model, but not in the univariate model. Higher ki67 was associated with a higher probability of pCR, but it had an inverse relation with survival when adjusted for pathological outcome.

Potential mechanisms for this finding must be investigated. One potential explanation is that NLR may reflect systemic inflammation. Some studies have suggested a detrimental role of neutrophils in immunological response to cancer [9, 10]. However, the relationship between peripheral neutrophil/lymphocyte counts and tumour-infiltrating lymphocytes (TILs) is unclear and some reported absence of association between peripheral blood count and tumour microenvironment [11].

Garcia-Torralba reported recently conflicting results regarding NLR role in early breast cancer [11]. The authors did not find a correlation between NLR and survival outcomes. However, patients included were treated both in adjuvant and neoadjuvant settings and TNBC comprised only 12% of the population. This raises the question whether NLR may be specifically relevant to the neoadjuvant scenario in TNBC but not across other subtypes of breast cancer or treatment settings. Some authors have also questioned whether NLR would have different behaviours according to race [3]. Our work comprised a significant proportion of black patients, who may have lower absolute neutrophil count than the white population [12]; although the median NLR did not differ among races in our cohort.

Previous literature used a cut-off point between 2 and 3 [4], which is consistent with the analysis in the present study. In a preliminary analysis from our group, using a receiver-operating characteristics curve, we considered cut-off point 2 as sufficient to maximise specificity for achieving pCR [7]. However, the best discrimination point is still to be determined, and exploratory analysis showed that different thresholds may be used. As a continuous variable, models that incorporate NLR could explore different cut-off points for different scenarios, as well as explore the relationship with other validated biomarkers, such as TILs.

Some recent studies have shown that high levels of NLR are associated with poorer survival across different metastatic tumours treated with checkpoint inhibitors [13–15]. However, it is not known the prognostic and predictive capacity of NLR in patients receiving neoadjuvant immunotherapy in TNBC. Further studies are necessary to evaluate NLR in this scenario and if it would be able to discriminate subpopulations and help tailor treatments.

pCR rates found in our study were lower than reported in the current literature of neoadjuvant therapies. Main reasons probably are the inclusion of higher risk population (63% of our cohort had clinical stage III disease; whereas, for example, 75.3% of patients had stage II disease in the phase III trial of pembrolizumab addition in neoadjuvant therapy [2]) and lack of platin in most regimens used, which has been demonstrated to increase pCR rates [16].

The first limitation of this study is its retrospective nature. Furthermore, some intrinsic bias cannot be controlled, as it was conducted entirely in the public health system in Brazil, with resource-limited treatment options, which did not include neoadjuvant immunotherapy, and adjuvant options with proven benefit in this scenario were not available during the period of the study (e.g., capecitabine and olaparib). Additionally, a few patients received platinum-containing regimens, which may prevent definite conclusions for this type of therapy. Finally, the absence of a validation dataset limits the generalisability of these findings. Our group is currently evaluating the role of the NLR in patients treated with the KEYNOTE-522 regimen within the Brazilian private healthcare system. This real-world data analysis, part of the Neo-Real study [17], will offer further validation of contemporary neoadjuvant strategies.

In conclusion, NLR is a readily available potential biomarker that can predict outcomes after NACT in TNBC. Further studies should aim at its utility for the selection of patients for escalation or de-escalation alternatives.

## Conflicts of interest

**G B P:** Financial support for educational programs and symposia: Libbs. **R C B:** Speaker fees and/or honoraria for consulting or advisory functions: Daiichi-Sankyo, Nestle Health Science, Addium, Gilead, MSD, BMS, AstraZeneca, Ache, Pfizer. Financial support for educational programs and symposia: AstraZeneca, Daiichi-Sankyo, MSD, Lilly. Institutional Research grant: Novartis, AstraZeneca.** L T:** Speaker fees and/or honoraria for consulting or advisory functions: Daiichi-Sankyo, MSD, AstraZeneca, Pfizer, Lilly, Novartis. Financial support for educational programs and symposia: AstraZeneca, Roche, Gilead. Institutional Research grant: Novartis. **R B S:** Speaker fees and/or honoraria for consulting or advisory functions: AstraZeneca, Daiichi-Sankyo, Eli Lilly, Gilead, Libbs, Pfizer, Novartis, MSD, and Roche. Financial support for educational programs and symposia: AstraZeneca, Daiichi-Sankyo, Gilead, Eli Lilly, and MSD. Institutional Research grant: AstraZeneca, Daiichi-Sankyo**. L K M, V V O, V R P, D C P, Y R S and L R C B** declare no conflicts of interest.

## Funding

The authors received no financial support for the research, authorship, and/or publication of this article.

## Author contributions

R C B, L T, G B P and R B S contributed to the study conception and design. G B P, L K M, V V O, V R P, D C P, Y R S and L R C B contributed to the conduct or collection. G B P, R C B, L T and R B S. contributed to data analysis and interpretation. G B P, R C B, L T and R B S contributed to the drafting of the manuscript and critical revisions. All authors gave their final approval of the manuscript to be submitted.

## Figures and Tables

**Table 1.** Patients characteristics.

	Total	NLR ≤ 2	NLR >2	*p*
Age (median, IQR)	48.5 (40.0–59.0)	51.0 (43.0–59.0)	47.0 (38.0–57.5)	<0.01
Clinical stage (*n*, %)				
II	254 (36.7%)	149 (42.7%)	105 (30.6%)	<0.01
III	438 (63.3%)	200 (57.3%)	238 (69.4%)	
*T* stage				
T0	3 (0.4%)	2 (0.6%)	1 (0.3%)	<0.01
T1a	1 (0.1%)	1 (0.3%)	0 (0%)	
T1b	2 (0.3%)	1 (0.3%)	1 (0.3%)	
T1c	6 (0.9%)	5 (1.4%)	1 (0.3%)	
T2	202 (29.2%)	120 (34.4%)	82 (23.9%)	
T3	267 (38.6%)	142 (40.7%)	125 (36.4%)	
T4a	13 (1.9%)	7 (2%)	6 (1.7%)	
T4b	142 (20.5%)	57 (16.3%)	85 (24.8%)	
T4c	12 (1.7%)	4 (1.1%)	8 (2.3%)	
T4d	43 (6.2%)	10 (2.9%)	33 (9.6%)	
Unknown	1 (0.1%)	0 (0%)	1 (0.3%)	
*N* stage				
N0	192 (27.7%)	107 (30.7%)	85 (24.8%)	0.1
N1	306 (44.2%)	158 (45.3%)	148 (43.1%)	
N2	141 (20.4%)	61 (17.5%)	80 (23.3%)	
N3	52 (7.5%)	23 (6.6%)	29 (8.5%)	
Unknown	1 (0.1%)	0 (0%)	1 (0.3%)	
Race (*n*, %)				
White	383 (55.3%)	185 (53%)	198 (57.7%)	0.34
Black	275 (39.7%)	148 (42.4%)	127 (37%)	
Others/Unknown	34 (4.9%)	16 (4.6%)	18 (5.2%)	
Grade (*n*, %)				
1	4 (0.6%)	2 (0.6%)	2 (0.6%)	0.35
2	245 (35.4%)	128 (36.7%)	117 (34.1%)	
3	421 (60.8%)	212 (60.7%)	209 (60.9%)	
Unknown	22 (3.2%)	7 (2%)	15 (4.4%)	
Ki67 (*n*, %)				
≥50%	534 (77.2%)	265 (75.9%)	269 (78.4%)	0.6
<50%	125 (18.1%)	68 (19.5%)	57 (16.6%)	
Unknown	33 (4.8%)	16 (4.6%)	17 (5%)	
NACT regimen (*n*, %)				
AC-T	597 (86.3%)	301 (86.2%)	296 (86.3%)	0.54
T-AC	73 (10.5%)	41 (11.7%)	32 (9.3%)	
Regimens with platinum	9 (1.2%)	3 (0.9%)	6 (1.8%)	
Others	13 (1.9%)	4 (1.1%)	9 (2.6%)	
NLR (median, IQR)	2 (1.5–2.75)	1.5 (1.17–1.7)	2.75 (2.29–3.67)	<0.01
Histology				
Invasive ductal carcinoma	615 (88.9%)	308 (88.3%)	307 (89.5%)	0.31
Metaplastic carcinoma	27 (3.9%)	19 (5.4%)	8 (2.3%)	
Micropapillary carcinoma	2 (0.3%)	0 (0%)	2 (0.6%)	
Lobular carcinoma	5 (0.7%)	2 (0.6%)	3 (0.9%)	
Apocrine carcinoma	11 (1.6%)	5 (1.4%)	6 (1.7%)	
Others/Unknown	32 (4.6%)	15 (4.3%)	17 (5%)	

**Table 2.** Logistic regression for prediction of pCR.

	Univariate analysis		Multivariable analysis	
	OR (95% CI)	*p* value	OR (95% CI)	*p* value
Age (<40 versus ≥40)	0.89 (0.59–1.31)	0.55	-	-
NLR (≥2 versus <2)	0.61 (0.43–0.85)	0.004	0.62 (0.44–0.89)	0.009
Clinical stage (III versus II)	0.52 (0.37–0.73)	<0.001	0.53 (0.37–0.76)	<0.001
Ki67 (≥50% versus <50%)	1.81 (1.14–3.00)	0.01	1.89 (1.17–3.13)	0.01

OR: Odds Ratio; 95% CI: 95% Confidence Interval

**Table 3.** Survival estimates according to different subgroups.

	EFS				OS			
	Univariate analysis		Multivariable analysis		Univariate analysis		Multivariable analysis	
	HR (95% CI)	*p* value	HR (95% CI)	*p* value	HR (95% CI)	*p* value	HR (95% CI)	*p* value
Age (<40 versus ≥40)	1.15 (0.88–1.49)	0.31	0.96 (0.72–1.29)	0.81	1.06 (0.79–1.42)	0.71	0.89 (0.64–1.25)	0.51
NLR (≥2 versus <2)	1.71 (1.33–2.18)	<0.001	1.43 (1.10–1.85)	0.007	1.76 (1.34–2.31)	<0.001	1.42 (1.06–1.90)	0.017
Clinical stage (III versus II)	2.67 (2.02–3.53)	<0.001	2.33 (1.70–3.18)	<0.001	3.17 (2.29–4.40)	<0.001	2.76 (1.92–3.98)	<0.001
Ki67 (≥50% versus <50%)	1.21 (0.90–1.64)	0.21	1.47 (1.05–2.07)	0.026	1.23 (0.88–1.72)	0.24	1.51 (1.03–2.20)	0.03
pCR status (No versus Yes)	5.66 (3.76–8.53)	<0.001	6.14 (3.74–10.08)	<0.001	5.47 (3.46–8.65)	<0.001	4.81 (2.88–8.05)	<0.001

EFS: Event-Free Survival; OS: Overall Survival; HR: Hazad Ratio; 95% Confidence Interval

## Supplementary Table

**Table S1.** EFS for different cut-off values.

Subgroup	NLR cutpoint	5 years EFS (95%CI) - high NLR	5 years EFS (95%CI) - low NLR	HR (95% CI)	*p* value
Clinical stage II	1.43	83% (74–94)	73% (66–81)	1.82 (0.91–3.61)	0.0841
Clinical stage II	1.76	82% (74–90)	71% (63–80)	2.04 (1.16–3.59)	0.0117
Clinical stage II	2	81% (74–89)	69% (60–80)	2.03 (1.18–3.47)	0.0085
Clinical stage II	2.5	80% (74–86)	63% (50–79)	2.4 (1.38–4.18)	0.0014
Clinical stage III	1.43	66% (55–79)	44% (39–50)	2.07 (1.36–3.15)	0.0005
Clinical stage III	1.76	59% (51–69)	43% (37–49)	1.7 (1.26–2.31)	0.0005
Clinical stage III	2	55% (48–63)	43% (37–51)	1.43 (1.08–1.89)	0.0111
Clinical stage III	2.5	55% (48–61)	38% (30–47)	1.71 (1.3–2.26)	0.0001
No pCR	1.43	64% (55–75)	43% (38–49)	2.06 (1.43–2.96)	0.0001
No pCR	1.76	59% (51–67)	41% (35–47)	1.81 (1.37–2.4)	<0.0001
No pCR	2	54% (48–62)	41% (35–48)	1.54 (1.19–1.99)	0.001
No pCR	2.5	54% (49–61)	34% (27–43)	1.86 (1.44–2.41)	<0.0001
pCR	1.43	98% (93–100)	85% (79–92)	6.09 (0.82–45.5)	0.0444
pCR	1.76	93% (88–99)	84% (76–92)	2.67 (0.97–7.36)	0.0476
pCR	2	92% (87–98)	82% (74–92)	2.23 (0.91–5.45)	0.0719
pCR	2.5	88% (82–94)	89% (79–100)	1.06 (0.35–3.17)	0.9189

**Table S2.** OS for different cut off values

Subgroup	NLR_cutpoint	5 years OS (95%CI) - low NLR	5 years OS (95%CI) - high NLR	HR (95% CI)	*p*_value
Clinical stage II	1.43	89% (81–98)	79% (73–87)	1.83 (0.81–4.14)	0.1422
Clinical stage II	1.76	87% (80–94)	78% (70–87)	1.91 (0.98–3.73)	0.0518
Clinical stage II	2	87% (80–93)	75% (66–86)	2.26 (1.2–4.29)	0.0099
Clinical stage II	2.5	85% (79–91)	72% (60–87)	2.52 (1.32–4.81)	0.0037
Clinical stage III	1.43	74% (64–85)	51% (46–58)	1.98 (1.25–3.12)	0.0028
Clinical stage III	1.76	67% (59–76)	50% (43–57)	1.72 (1.23–2.41)	0.0013
Clinical stage III	2	62% (55–70)	50% (44–58)	1.44 (1.06–1.95)	0.0194
Clinical stage III	2.5	62% (56–69)	44% (36–54)	1.74 (1.29–2.34)	0.0002
No pCR	1.43	74% (65–84)	52% (46–58)	2.03 (1.35–3.05)	0.0005
No pCR	1.76	68% (60–76)	50% (44–56)	1.81 (1.33–2.48)	0.0002
No pCR	2	64% (57–71)	50% (43–57)	1.59 (1.19–2.13)	0.0014
No pCR	2.5	64% (58–70)	42% (35–52)	1.95 (1.47–2.59)	<0.0001
pCR	1.43	98% (93–100)	84% (78–91)	5.58 (0.74–41.79)	0.0592
pCR	1.76	93% (86–99)	83% (75–92)	2.39 (0.86–6.63)	0.0853
pCR	2	91% (85–97)	82% (73–93)	2 (0.8–4.98)	0.1278
pCR	2.5	87% (80–93)	89% (79–100)	1.12 (0.37–3.38)	0.8412
